# Simulation of Quantum Dynamics Based on the Quantum Stochastic Differential Equation

**DOI:** 10.1155/2013/424137

**Published:** 2013-05-28

**Authors:** Ming Li

**Affiliations:** School of Automation, Guangdong University of Technology, No. 100 Waihuan Xi Road, Guangzhou Higher Education Mega Center, Pan Yu District, Guangzhou, Guangdong 510006, China

## Abstract

The quantum stochastic differential equation derived from the Lindblad form quantum master equation is investigated. The general formulation in terms of environment operators representing the quantum state diffusion is given. The numerical simulation algorithm of stochastic process of direct photodetection of a driven two-level system for the predictions of the dynamical behavior is proposed. The effectiveness and superiority of the algorithm are verified by the performance analysis of the accuracy and the computational cost in comparison with the classical Runge-Kutta algorithm.

## 1. Introduction

Since Nelson successfully described the kinematics law of the quantum fluctuations by the Itô equation [1] and the Schrödinger equation was derived from Newtonian mechanics; the stochastic interpretation of quantum mechanics was established, in which a diffusion process was used to analyze the quantum fluctuation instead of the wave function. Then, the stochastic mechanics has gradually drawn much attention with research fields ranging from atomic and optical physics to condensed matter physics and quantum information science [2]. It becomes clear that a deep understanding of the effects of environments on a quantum system such as the mechanisms of decoherence and the dynamics of entanglement in the framework of quantum open systems is both of fundamental interest in quantum foundation issues and of practical importance in quantum information sciences. Many scholars have made thorough research on the quantum diffusion movement based on the basic theory and achieved fruitful results. For example, the normative structure of the dynamics equation of the particle fluctuation and its stability analysis methods were determined, followed by the unified interpretation of the Brown motion and the basic equation of the quantum mechanics [3–5]. The quantum stochastic dynamics elaborated the organic link between the microscopic behavior and macroevolution of the system, by which the details of the system evolution from any initial state to the final state can be analyzed. It has been successfully applied to the proliferation of microscopic particles, the molecular motors, the quantum chaos, and so forth [6].

Though the deterministic differential equations of quantum stochastic mechanics are relatively complex, it can be seen that with the development of the computer technology [7], the evolution of a microsystem can be analyzed using the numerical simulation method [8–10]. The quantum trajectory method is a typical one. It can be used for a wide range of open quantum systems to solve the master equation by unraveling the density operator evolution into individual stochastic trajectories in Hilbert space [11]. Over the last twenty years the theory of quantum trajectories has been developed by many researchers for a variety of purposes, including modeling continuously monitored open systems, improving numerical calculation and investigating the problem of quantum measurement [12, 13].

In this paper, based on the quantum stochastic dynamics, the master equation describing the time evolution law of the quantum state and its reduced density operator are investigated, and the effect of nonunitary operators on the evolution of the system is analyzed. Then the quantum stochastic differential equation is established to describe the microkinetic characteristics of the system, and a numerical iterative algorithm for the simulation of the system evolution is proposed. The practicality and advantages of the algorithm are verified by comparison with the classical Runge-Kutta numerical iterative algorithm, which is followed by further discussions on the convergence of the algorithm.

## 2. Methods

The quantum state diffusion theory replaces the deterministic evolution of the density operator *ρ* representing an ensemble of open systems [14]

(1) $$\begin{array}{c} \dot{\rho }=-\frac{i}{\hbar }\left[H,\rho \right]+\sum_{j}\left(L_{j}\rho L_{j}^{+}-\frac{1}{2}L_{j}^{+}L_{j}\rho -\frac{1}{2}\rho L_{j}^{+}L_{j}\right) \end{array}$$

by a unique stochastic diffusion of a quantum state, representing an individual system of the ensemble in interaction with its environment. *H* is the Hamiltonian, and *L*_*j*_ are a set of environment operators which represent the collective effects of interaction with the environment.

However, for some complicated systems, it can be very difficult to get either the analytical or the numerical solution of (1). In that case, it is often advantageous to take alternative ways considering an unraveling of the master equation into individual quantum trajectories. Quantum state diffusion (QSD) is one of these unraveling techniques. The corresponding quantum state diffusion equation is a stochastic differential equation for the normalized state vector |*ψ*〉 representing the pure state of the system that evolves according to the QSD equation. The differerential Itô form is [15]

(2) |dψ〉=−iℏH|ψ〉+∑j(〈Lj+〉Lj−12Lj+Lj−12〈Lj+〉〈Lj〉)|ψ〉dt+∑j(Lj−〈Lj〉)|ψ〉dξj,

where 〈*L*_*j*_〉 are defined by

(3) $$\begin{array}{c} \langle L_{j}\rangle =\langle \psi |L_{j}|\psi \rangle . \end{array}$$

In the QSD equation, the standard normalized terms d*ξ*_*j*_ represent independent complex Wiener processes and satisfy the relations

(4) $$\begin{array}{c} E\text{d}\xi_{j}=0,\;\;E\left(\text{d}\xi_{j}\text{d}\xi_{j^{\prime }}\right)=0,\\ E\left(\text{d}\xi_{j}^{*}\text{d}\xi_{j^{\prime }}\right)=\delta_{jj^{\prime }}\text{d}t, \end{array}$$

where **E**(·) denotes an ensemble average of the noise. QSD reproduces the master equation in the mean

(5) $$\begin{array}{c} E\left(|\psi \rangle \langle \psi |\right)=\rho . \end{array}$$

That is to say, the reduced state of the system, *ρ*, is obtained as an ensemble average. And this is what is meant by an unraveling of the master equation. Expectation values for operators obey a similar relationship:

(6) $$\begin{array}{c} {\langle \hat{O}\rangle }_{\rho }=\mathrm{Tr}\left\{\hat{O}\rho \right\}=E\left({\langle \hat{O}\rangle }_{\psi }\right). \end{array}$$

The use of QSD as a practical algorithm to solve master equations has been widely investigated [16, 17]. This includes calculations of output spectra in quantum optics [18]. As a practical method of computation, QSD gains over the direct solution of the master equation, because of a basis of *N* states, QSD needs a computer store with *N* elements, and the time of computation is also proportional to *N*. For the direct solution these are proportional to *N* ^2^. 

In this paper, we take advantage of the system simulation method to simulate the evolutionary behavior of the open quantum systems and thus calculate and analyze various physical properties of the ensemble of open quantum systems. However, noting that we cannot usually get the analytical solution of (2), an alternative way is to find the numerical solution of the system evolution and investigate various control algorithms and control strategies based on the simulation method, which is a powerful tool built on the systems science, system identification, control theory, and computer technology for the analysis and synthesis of complex systems, especially large-scale systems [19].

In the system simulation, we should pay attention to the problem that the physical description of the stochastic process is relied on the master equation in the Lindblad operator form. If we want to get the numerical solution, the Lindblad operator must be explicitly quantified. Fortunately, existing results have summarized various forms of decoherence Lindblad operator in open quantum systems for reference; a general form of (2) can be written as [20]

(7) $$\begin{array}{c} \text{d}\psi \left(t\right)=D_{1}\left(\psi \left(t\right)\right)\text{d}t+D_{2}\left(\psi \left(t\right)\right)\text{d}W\left(t\right) \end{array}$$

in which d*W*(*t*) is a Wiener incremental process and the random items characterizing the system decoherence are

(8) $$\begin{array}{c} D_{1}=\left(\langle L^{+}\rangle L-\frac{1}{2}L^{+}L-\frac{1}{2}\langle L^{+}\rangle \langle L\rangle \right)|\psi \rangle , \end{array}$$

(9) $$\begin{array}{c} D_{2}=\left(L-\langle L\rangle \right)|\psi \rangle . \end{array}$$

According to the above system model, in the given interval [0, *t*_*f*_], a sample of realization can be generated by the following algorithm [20].

(1) At the initial time *t* = 0, the initial state of the process *ψ* ^*r*^(0) is determined by the initial distribution.
(2) It is assumed that at time *t*, the normalized state *ψ* ^*r*^(*t*) is reached through a quantum jump; then we set $\psi^{r}(t)=\tilde{\psi }$.
(3) Determine a random waiting time *τ*. This can be done, for example, by drawing a random number *η* which is uniformly distributed over the interval [0,1] and by determining *τ* from the equation:
  (10) $$\begin{array}{c} \eta =1-F\left[\tilde{\psi },\tau \right]={||e^{-i\hat{H}\tau }\tilde{\psi }||}^{2}. \end{array}$$
 First we define the defect of the waiting time distribution *q* by the identity
  (11) $$\begin{array}{c} q\equiv \underset{\tau \to \infty }{\lim}{||\exp\left(-i\hat{H}\tilde{\psi }\right)||}^{2}. \end{array}$$

 For *η* > *q*, a unique solution can be obtained. If *η* ≤ *q*, we set *τ* = *∞* in which case there will be no further jumps. Within the time interval [*t*, *t* + *τ*] the realization follows the deterministic time evolution:
  (12) $$\begin{array}{c} \psi^{r}\left(t+s\right)=\frac{e^{-\text{i}Hs}\tilde{\psi }}{||e^{-\text{i}Hs}\tilde{\psi }||},\;0\leq s\leq \tau . \end{array}$$
(4) At time *t* + *τ* (if *τ* is finite and *t* + *τ* < *t*_*f*_), one of the possible jumps labeled by the index *i* occurs according to
  (13) dψ(t)=−i(H^+i2∑iγi||Liψ(t)||2)ψ(t)dt+∑i(Liψ(t)||Liψ(t)||−ψ(t))|ψ〉dNi(t).
 Then we select a specific jump of type *i* with probability
  (14) $$\begin{array}{c} p_{i}=\frac{\gamma_{i}{||L_{i}\psi^{r}\left(t+\tau \right)||}^{2}}{\sum_{i}\gamma_{i}{||L_{i}\psi^{r}\left(t+\tau \right)||}^{2}}. \end{array}$$
 And then we update the state of system as
  (15) $$\begin{array}{c} \psi^{r}\left(t+\tau \right)=\frac{L_{i}\psi^{r}\left(t+\tau \right)}{||L_{i}\psi^{r}\left(t+\tau \right)||}. \end{array}$$
(5) Repeat steps (1) to (4) until the desired final time *t*_*f*_ is reached, which yields the realization *ψ* ^*r*^(*t*) over the whole time interval [0, *t*_*f*_]. Once a sample of realizations *ψ* ^*r*^(*t*),  *r* = 1,2, 3,…, *R*, has been generated according to this algorithm, any statistical quantity can be estimated through an appropriate ensemble average.

According to the above framework, an iterative algorithm is described as follows:

(16) ψk+1=ψk+12(D1(ψ~k)+D1(ψk))Δt+14(D2(ψk+)+D2(ψk−)+2D2(ψk))ΔWk+14(D2(ψk+)−D2(ψk−)){(ΔWk)2−Δt}Δt−1/2,

where

(17) $$\begin{array}{c} {\tilde{\psi }}_{k}=\psi_{k}+D_{1}\left(\psi_{k}\right)\Delta t+D_{2}\left(\psi_{k}\right)\Delta W_{k},\\ {\tilde{\psi }}_{k}^{\pm }=\psi_{k}+D_{1}\left(\psi_{k}\right)\Delta t\pm D_{2}\left(\psi_{k}\right)\sqrt{\Delta t}. \end{array}$$

## 3. Results and Discussion

We consider the process describing the direct photodetection of a driven two-level system, and the piecewise deterministic process is given by the following equation [21, 22]:

(18) dψ(t)=−i(H^+iγ2||σ−ψ(t)||2)ψ(t)dt+(σ−ψ(t)||σ−ψ(t)||−ψ(t))dN(t).

The Poisson increments d*N*(*t*) satisfy

(19) $$\begin{array}{c} \hat{H}=H-\frac{\text{i}}{2}\sum_{i}\gamma_{i}L_{i}^{†}L_{i},\\ \text{d}N{\left(t\right)}^{2}=\text{d}N\left(t\right),\\ E\left[\text{d}N\left(t\right)\right]={||\sigma_{-}\psi \left(t\right)||}^{2}\text{d}t. \end{array}$$

The corresponding stochastic Schrodinger equation takes the form

(20) dψ(t)=−iHLψ(t)dt+γ2(〈σ−+σ+〉ψσ−−σ−σ+    −14〈σ−+σ+〉ψ2)ψ(t)dt+γ(σ−−12〈σ−+σ+〉ψ)ψ(t)dW(t),

where

(21) $$\begin{array}{c} H_{L}=-\frac{\Omega }{2}\left(\sigma_{-}+\sigma_{+}\right). \end{array}$$

In the numerical simulations, it is assumed that the atom is in its ground state |*g*〉 and the probability of finding the atom in the excited state |*e*〉 can be calculated as follows:

(22) $$\begin{array}{c} \rho_{11}\left(t\right)=\langle e\left|\rho \left(t\right)\right|e\rangle . \end{array}$$

From a sample of realizations this probability is estimated by determining the average

(23) $$\begin{array}{c} {\hat{M}}_{t}=\frac{1}{R}\sum_{r=1}^{R}{\left|\langle e\psi^{r}\left(t\right)\rangle \right|}^{2}. \end{array}$$

An appropriate estimator for the corresponding statistical errors in the determination of ${\hat{M}}_{t}$ from a finite sample of size *R* is given by

(24) $$\begin{array}{c} {\hat{\sigma }}_{t}^{2}=\frac{1}{R\left(R-1\right)}\sum_{r=1}^{R}{\left({\left|\langle e\psi^{r}\left(t\right)\rangle \right|}^{2}-{\hat{M}}_{t}\right)}^{2}. \end{array}$$

According [21, 22], the analytical solution of the process is

(25) $$\begin{array}{c} \rho_{11}\left(t\right)=\frac{\Omega^{2}}{\gamma_{0}^{2}+2\Omega^{2}}\left[1-e^{-3\gamma_{0}t/4}\left\{\cos\mu t+\frac{3\gamma_{0}}{4\mu }\sin\mu t\right\}\right], \end{array}$$

where

(26) $$\begin{array}{c} \mu =\sqrt{\Omega^{2}-{\left(\frac{\gamma }{4}\right)}^{2}}. \end{array}$$

Hence, it is possible to compare the numerical results with the analytical results. 

### 3.1. Simulation Results

**Figure 1:**
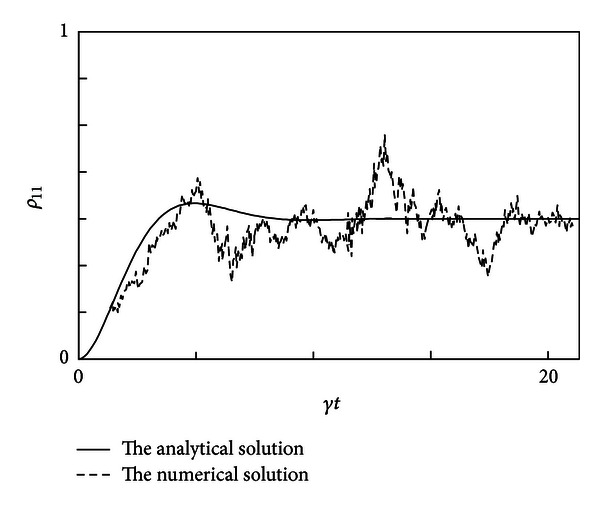
The comparison between the numerical solution of *ρ*_11_ and the analytical solution direct photodetection of a driven two-level system with the parameters: Δ*t* = 0.01, *Ω* = 0.45, and *γ* = 0.3.

 A sample of realizations for *ρ*_11_ calculated from (20) is as the dashed line shows in Figure 1 with the following parameters: step size Δ*t* = 0.01, *Ω* = 0.45, and *γ* = 0.3, while the smooth line gives the analytical solution.

In order to discuss the performance of the algorithm, we introduced the classic Runge-Kutta iterative algorithm for generating the sample of realizations as follows [23]:

(27) ψk+1=ψk+16{ψk1+2ψk2+2ψk3+ψk4}Δt+D2(ψk)ΔWk,

where

(28) $$\begin{array}{c} \psi_{k}^{1}=D_{1}\left(\psi \left(k\right)\right),\\ \psi_{k}^{2}=D_{1}\left(\psi_{k}+\frac{1}{2}\Delta t\psi_{k}^{1}\right),\\ \psi_{k}^{3}=D_{1}\left(\psi_{k}+\frac{1}{2}\Delta t\psi_{k}^{2}\right),\\ \psi_{k}^{4}=D_{1}\left(\psi_{k}+\Delta t\psi_{k}^{3}\right). \end{array}$$

In this paper, the computer simulation platform is the Intel(R) Core (TM)2 Duo CPU E7500 @ 2.93 GHz under the Windows XP operating system with the numerical calculation software Matlab7. By selecting different simulation step sizes Δ*t*, we can get set of results according to the corresponding Δ*t* with different algorithms:

1. the estimated means of |〈*e*|*ψ* ^*r*^(*T*)〉|^2^ minus the exact values obtained by the analytical solution of *ρ*_11_(*t*) for different step sizes and methods, (|〈*e*|*ψ* ^*r*^(*T*)〉|^2^ − *ρ*_11_(*t*)), as Table 1 shows,
2. the normalized CPU time versus the absolute error for the data points, as Table 2 shows.

As can be seen from Table 1, when the simulation step size is increased, there is little change in the errors in the proposed algorithm, while the errors using the Runge-Kutta method increase linearly. That is, in a certain step length range, the proposed iterative algorithm can generate more accurate approximation numerical solution than the Runge-Kutta algorithm in comparison to the analytical solution.

At the same time, we can draw a conclusion from Table 2: when obtaining a more accurate numerical solution using the proposed algorithm, the computational time is on the same order of magnitude as the Runge-Kutta method consumes. And it is not difficult to find that, when the step length is gradually reduced, accompanied by improving the accuracy, the computational time of the Runge-Kutta algorithm grows faster.

Taking the above two advantages comparing to the classical Runge-Kutta algorithm, the proposed algorithm can generate a more accurate sample of realizations while paying lower computational costs, which reflects the superiority and practicality of the proposed algorithm.

### 3.2. Convergence Analysis

Generally speaking, the smaller the step size Δ*t* is, the closer the numerical solution matches the true solution, and consequently the convergence seems to take place. We denote *X*(*t*_*k*_) as the true analytical solution and *X*_*k*_ as the numerical approximation. Noting that *X*(*t*_*k*_) and *X*_*k*_ are random variables, we can measure the difference using **E**(*X*_*k*_ − *X*(*t*_*k*_)), where **E**(·) represents the expected value. Then a method is said to have strong order of convergence equal to *β* if there exists a constant *M* such that

(29) $$\begin{array}{c} E\left|X_{k}-X\left(t_{k}\right)\right|\leq M\Delta t^{\beta } \end{array}$$

for any fixed *t*_*k*_ ≡ *t*_0_ + *k*Δ_*t*_ ∈ [0, *t*_*f*_] and Δ*t* sufficiently small.

The strong order of convergence (29) measures the rate at which the “mean of the error” decays as Δ*t* → 0. A less demanding alternative is to measure the rate of decay of the “error of the means.” This leads to the concept of weak convergence. A method is said to have weak order of convergence equal to *β* if there exists a constant *M* such that for all functions *p* in some class

(30) $$\begin{array}{c} \left|E_{p}\left(X_{k}\right)-E_{p}X\left(t_{k}\right)\right|\leq M\Delta t^{\beta } \end{array}$$

for any fixed *t*_*k*_ ≡ *t*_0_ + *k*Δ_*t*_ ∈ [0, *t*_*f*_] and Δ*t* sufficiently small [24].

According the above theory, we consider a numerical algorithm to integrate (7) in the time period [*t*_0_, *t*_0_ + *T*]. Such an algorithm will generate a discrete time approximate realizations *ψ*_*k*_ for the exact process *ψ*(*t*_*k*_) at the given time *t*_*k*_ ≡ *t*_0_ + *k*Δ_*t*_, where *k* = 0,1,…, *n* = *T*/Δ*t*. Before our discussion, several conventions should be made as follows.

1. In this section, *ψ*_*k*_ always represents a numerical approximation, while *ψ*(*t*_*k*_) stands for the exact process described by (7).
2. Similarly, we define the discrete time approximation of the density matrix as *ρ*_*k*_ = **E**[|*ψ*_*k*_〉〈*ψ*_*k*_|], whereas *ρ*(*t*) = **E**[|*ψ*(*t*)〉〈*ψ*(*t*)|] is the exact density matrix satisfying the Lindblad equation  (9).
3. We always set a deterministic initial state *ψ*_0_ ≡ *ψ*(*t*_0_).

In order to illustrate the numerical convergence of the algorithm, we will compare the Taylor expansion of the exact density matrix *ρ*(*t*) which is given by (31) with the generated approximation *ρ*_*k*_:

(31) $$\begin{array}{c} \rho \left(t+\Delta t\right)=\rho \left(t\right)+L\rho \left(t\right)\Delta t+\frac{1}{2}L^{2}\rho \left(t\right)\Delta t^{2}+O\left(\Delta t^{3}\right). \end{array}$$

We compare the numerical simulation approximate obtained by the proposed algorithm to the true evolution *ρ*(*t*). The single-step error of a certain numerical scheme may then be expressed through the difference

(32) $$\begin{array}{c} \rho_{1}-\rho \left(t_{1}\right)=O\left(\Delta t^{\gamma }\right) \end{array}$$

which means that the strategy reproduces the Taylor expansion of *ρ*(*t*) including terms of order *γ* − 1 in Δ*t*.

Thus, it is direct to prove that the integration over a finite time period [*t*_0_, *t*_0_ + *T*] decreases the convergence order by one since *n* = *T*/Δ*t* time steps are needed to compute the density at time *t*_*n*_ = *t*_0_ + *T*, that is,

(33) $$\begin{array}{c} \rho_{n}-\rho \left(t_{n}\right)=O\left(\Delta t^{\beta }\right) \end{array}$$

with *β* = *γ* − 1. If (33) can be satisfied, the numerical strategy is defined to be a strategy of order *β* [15]. It should be noted that (33) is a special case of weak convergence of order *β* describing the degree of proximity of the probability distributions of *ψ*_*n*_ and *ψ*(*t*_*n*_) which is a much weaker criterion comparing to strong convergence defined in (29). In actual applications, one tends to care about this weaker form of convergence especially when considering the approximation of functionals of the stochastic variable.

When investigating the stochastic differential equations and numerical simulation solution process, if the issues are related to the numerical simulation of the stochastic process, the evaluation criteria of the solutions convergence are usually defined as the numerical approximation curve. It must be sufficiently close to the real trace of the evolution. That is to say, the higher the convergence order is, the closer the distribution of the numerical solution is with the analytical solution of the distribution. So it requires that the probability distribution *ψ*_*n*_ obtained by the simulation iterative algorithm is close enough to the probability distribution determined by the quantum master equation [24]. 

Performing the error analysis according to (16) one can find it converging with order *β* = 2 in contrast to the classical Runge-Kutta method with *β* = 1. Thus, it is really a higher-order strategy in the weaker convergence sense.

## 4. Conclusions

The decoherence of open quantum systems usually makes the system evolve from the initial pure state to mixed states (in some cases, may also be mixed state into a pure state). Being a powerful tool for investigating the open quantum systems, the quantum master equation can give a quantitative description of the transition, dissipation, and decoherence caused by the interaction between the closed system and the environment. Taking this as the starting point of our research, in order to obtain the evolution of the open quantum systems according to its dynamic characteristics, we used the system simulation method to get the numerical solution to the reduced density operator of a typical open quantum system. And its effectiveness and superiority were verified in comparison with the classical algorithm. Further research includes the control scheme [25, 26] for quantum manipulation based on the characteristics of quantum dynamics.

## Figures and Tables

**Table 1:** The estimated means of |〈*e*|*ψ* ^*r*^(*T*)〉|^2^ minus the exact values obtained by the analytical solution of *ρ*_11_(*t*), that is, (|〈*e*|*ψ* ^*r*^(*T*)〉|^2^ − *ρ*_11_(*t*)), for different step sizes Δ*t* and methods.

	0.01	0.02	0.05	0.10	0.15	0.20	0.25
The proposed algorithm	0.00012	0.00018	0.00015	0.00025	0.00021	0.00017	0.00021
Runge-Kutta	0.00026	0.00049	0.00070	0.00121	0.00142	0.00220	0.00311

**Table 2:** The normalized CPU time for different step sizes Δ*t* and methods.

	0.01	0.02	0.05	0.10	0.15	0.20	0.25
The proposed algorithm	0.153	0.108	0.081	0.061	0.050	0.038	0.007
Runge-Kutta	1	0.162	0.100	0.083	0.072	0.065	0.030
